# Anaphylaxis Preparedness among Preschool Staff before and after an Educational Intervention

**DOI:** 10.1155/2015/231862

**Published:** 2015-08-02

**Authors:** Ashley A. Foster, Ronna L. Campbell, Sangil Lee, Jana L. Anderson

**Affiliations:** ^1^Department of Emergency Medicine, University of California, San Francisco, San Francisco, CA, USA; ^2^Department of Emergency Medicine, Mayo Clinic, Rochester, MN, USA; ^3^Department of Emergency Medicine, Mayo Clinic Health System in Mankato, Mankato, MN, USA

## Abstract

*Introduction*. Children with severe food allergies may spend many hours in the preschool setting. Little is known about anaphylaxis recognition and management preparedness among preschool staff. The objective of this study was to assess anaphylaxis preparedness among preschool staff. *Methods*. Anonymous questionnaires were administered before and after a 40-minute educational seminar on anaphylaxis recognition and management. *Results*. In total, 181 individuals participated in the preintervention survey and 171 participated in the postintervention survey. The comfort level with recognizing anaphylaxis and administering an epinephrine autoinjector significantly increased after the intervention (*P* < .001 for both). Of the 5 steps needed to administer an epinephrine autoinjector, staff named a mean (SD) of 3 (1.3) steps in the correct order compared with 4.2 (1.1) steps after the educational intervention (*P* < .001). *Conclusion*. This study shows that a brief education intervention can significantly increase caregiver comfort regarding identifying anaphylaxis and administering an epinephrine autoinjector.

## 1. Introduction

Anaphylaxis is defined as a rapid-onset, potentially fatal allergic reaction [1]. Food allergy remains the most common cause of anaphylaxis in children [2]. Although the true incidence and prevalence rates of pediatric anaphylaxis are unknown because of differing clinical definitions of anaphylaxis, a recent population-based study in Rochester, Minnesota, by Decker et al. [3] showed the highest age-specific incidence in children aged 0 to 9 years. Accidental exposure to an allergen is frequent in the pediatric population, with up to 50% of children with a known food allergy having an accidental exposure in a 1-year period [4]. Because children spend much of their time in a school environment, potential exposure to allergens and subsequent anaphylactic reactions are anticipated at many preschools and schools [5]. Nevertheless, recent studies have shown that few preschool staff are trained to administer epinephrine, and those who have received training are still often unable to properly administer the appropriate therapy [6, 7].

Although preventing allergic reactions to known triggers remains the best management strategy, in the case of severe allergy or anaphylaxis, epinephrine is the first-line treatment [8]. Anaphylaxis requires quick action to prevent complications and potential fatality [9]. Studies show that delayed treatment with epinephrine is a major risk factor for death from anaphylaxis [9–11]. Although the universally recommended method of treating anaphylaxis is intramuscular injection of epinephrine, it still is uncommonly used as therapy for food-induced anaphylaxis in the prehospital setting or emergency department [12].

Because children with severe food allergies can spend many hours in the preschool setting, childcare staff must be proficient in recognizing anaphylaxis and using an epinephrine autoinjector [13]. Currently, many preschools do not have a nurse on staff who could quickly treat a child in anaphylaxis; thus, classroom teachers are responsible for taking action and administering an epinephrine autoinjector when needed. However, deficits exist in staff education regarding recognition of symptoms of food allergy and knowledge about the correct use of an epinephrine autoinjector during anaphylaxis [7, 14]. Although recognition and management of anaphylaxis has been studied in preschool directors [13, 15], little is known about whether preschool teachers and other staff members can recognize anaphylaxis and properly use an epinephrine autoinjector. Thus, the objective of this study was to assess anaphylaxis preparedness among preschool staff members, including school directors, teachers, teaching assistants, and ancillary staff, before and after an educational intervention.

## 2. Methods

This project was approved by our institutional review board.

### 2.1. Participants

We identified preschools with children aged 6 years and under in the community. Directors were contacted via telephone and invited to participate in the study between September 2011 and May 2012.

Participating staff included preschool directors, teachers, assistants, teaching aides, and home visitors. Participation was voluntary. Staff who could not attend the educational session completed only the preintervention questionnaire. Consent was obtained from all preschool directors via telephone, and consent from the remaining staff was obtained before survey administration.

### 2.2. Intervention and Questionnaire

Preschool staff attended a 40-minute educational seminar (presented by Ashley A. Foster) that included an overview of recognition and immediate management of anaphylaxis, a hands-on demonstration, and practice session with an epinephrine autoinjector training device.

Anonymous questionnaires were administered to staff before and after the educational session to assess baseline characteristics and the effectiveness of the intervention (the same questionnaires were used both times). The postintervention questionnaire was administered immediately after the educational seminar.

The survey assessed staff characteristics, educational experience and responsibilities, training in and comfort level associated with anaphylaxis recognition, competence and comfort level of epinephrine autoinjector administration, and barriers to epinephrine administration. Comfort levels associated with recognizing anaphylaxis and administering an epinephrine autoinjector were evaluated on a scale of 1 to 10, with 1 indicating not comfortable and 10 indicating very comfortable. Participants were asked to sequentially order the steps of epinephrine autoinjector administration in the setting of anaphylaxis. Steps included (1) make a fist around the epinephrine autoinjector; (2) remove the protective cap; (3) push the injector firmly against child's anterior-lateral thigh; (4) hold for 10 seconds; and (5) discard epinephrine autoinjector in the proper container. Potential barriers to successful recognition and administration of epinephrine were assessed.

### 2.3. Statistical Analysis

Data are presented as mean (SD). Categorical data are presented as percent frequency of occurrence. Comparisons between features of interest were evaluated using Spearman rank correlation coefficients and the Kruskal-Wallis, Wilcoxon rank sum, *χ* ^2^, and Fisher exact tests.

## 3. Results

We contacted 24 preschools and 10 (42%) agreed to participate. The preintervention survey was completed by 181 staff members, and 171 participated in the educational seminar and postintervention survey. Staff characteristics, educational experience, and responsibilities are summarized in Table 1. Eighty-seven participants (51%) had fewer than 5 years of teaching experience. Although 120 participants (70%) reported prior training using an epinephrine autoinjector, only 98 (57%) reported prior training on anaphylaxis recognition. The majority of those who answered the survey (*n* = 151 (90%)) indicated wanting more anaphylaxis education.

Survey responses, stratified by participant educational role, are summarized in Table 2. We observed no significant differences in preintervention comfort level with recognizing anaphylaxis or with administering an epinephrine autoinjector on the basis of educational role. Potential barriers to successful recognition and administration of epinephrine were identified before the educational intervention (Table 2). The most common perceived barriers were uncertainty about anaphylaxis recognition (91/132 (69%)) and uncertainty about using the epinephrine autoinjector (71/132 [54%]).

The 5 steps to administering an epinephrine autoinjector were assessed (Table 3). Before the educational intervention, staff ordered a mean (SD) of 3 (1.3) steps correctly, whereas they correctly ordered 4.2 (1.1) steps correctly after the educational intervention (*P* < .001). Overall, 98 staff members (57%) ordered all 5 steps correctly after the intervention compared with only 28 (15%) before the intervention. The number of correctly identified epinephrine autoinjector administration steps was weakly but positively correlated with comfort level with epinephrine administration (Spearman rank correlation coefficient, 0.16; *P* = .04).

Comfort levels with recognizing anaphylaxis and administering an autoinjector were evaluated, and responses before and after the educational intervention are summarized in Table 3. Comfort level with recognizing anaphylaxis significantly increased after the education session (mean (SD), 5.2 (2.4) versus 8.7 (1.2); *P* < .001). Two participants (1%) indicated that they were less comfortable recognizing anaphylaxis after the seminar, 19 participants (11%) had the same level of comfort, and the remaining 150 participants (88%) had a higher level of comfort. Additionally, the comfort level with administering an epinephrine autoinjector significantly increased after the intervention (mean (SD) increase, 3.7 (2.3); *P* < .001). Almost all participants (99%) believed that they benefited from the seminar, with 126 (76%) endorsing education annually.

Prior training affected preintervention comfort levels regarding preparedness. Individuals who received prior anaphylaxis recognition training had significantly higher preintervention anaphylaxis recognition comfort levels compared with participants who had not received such training (mean: 5.7 versus 4.3; *P* < .001). Similarly, participants who received prior training in epinephrine autoinjector administration had a significantly higher comfort level with administering an autoinjector in the event of anaphylaxis than those without previous training (mean: 5.9 versus 4.0; *P* < .001).

## 4. Discussion

An increasing incidence of food allergies and anaphylaxis, especially in children aged 5 years and younger, has been reported in multiple countries, including the United States, United Kingdom, and Australia [16]. Because intramuscular epinephrine is the definitive therapy for anaphylaxis, it is imperative to establish a knowledge base and increase preparedness among preschool staff regarding recognition of anaphylaxis and the use of an epinephrine autoinjector [7, 13, 15, 17]. To our knowledge, this is the first study to assess anaphylaxis recognition and epinephrine administration preparedness among multiple categories of preschool staff (e.g., school directors, teachers, teaching assistants, ancillary staff).

Because teachers are more likely to be directly supervising the students throughout the day, teachers specifically must be able to rapidly recognize and appropriately manage anaphylaxis. Our results showed that although 64% of all teachers reported previous training in anaphylaxis recognition and 80% had prior training on epinephrine autoinjector administration, 70% were uncertain about recognizing anaphylaxis and considered it a barrier to epinephrine administration. Further, more than half were uncertain about how to administer epinephrine.

Because the ability to recognize a severe allergic reaction is the critical first step in managing anaphylaxis, health care providers should focus attention not only on education about proper autoinjector technique but also on the clinical signs and symptoms of a child in anaphylaxis. A study by Bansal et al. [13] reported that approximately 50% of preschool directors had prior training on identification and treatment of an allergic reaction. Overall, 57% of our participants had received prior training on recognizing anaphylaxis and 71% reported prior training in administering an epinephrine autoinjector.

An important outcome of anaphylaxis education is assessment of the comfort level of preschool staff who will be administering the epinephrine autoinjector in the event of pediatric anaphylaxis. We reported a significant increase in comfort level of recognizing anaphylaxis and administering an epinephrine autoinjector (*P* < .001 for both) when comparing questionnaire responses before and after the education seminar. In our study, one of the most common barriers to autoinjector administration was the lack of comfort in knowing how to use the autoinjector. To our knowledge, no previous study has specifically assessed comfort levels in a preschool staff population and showed a significant increase in comfort for both the recognition of anaphylaxis and the technique for administering epinephrine. Our study indicates that staff had similarly improved comfort levels after the education seminar, regardless of their prior training.

Two participants noted decreased comfort in recognition of anaphylaxis in the postintervention questionnaire. Possible explanations include an overwhelming amount of information in the seminar or a need for different educational mediums to better communicate the information included in the seminar.

Finally, participants in this study showed an increase in correctly sequenced steps for epinephrine autoinjector administration after the intervention. These findings are similar to those of Bansal et al. [13], who reported that, after an allergy seminar, 77% of center directors surveyed would administer intramuscular epinephrine correctly compared with only 24% before the educational seminar.

## 5. Limitations

Preschools in the area were approached and asked to participate, irrespective of the number of students and faculty, public versus private status, or prior anaphylaxis training. Consequently, those who agreed to participate in the study may have had a special interest in anaphylaxis in children. The issue with selection bias needs to be considered carefully, as reported by Nguyen Luu et al. [18]. Additionally, although personal demographics of participants were not collected, the participant population was relatively homogenous (e.g., white, middle aged, predominantly female).

Limitations of this study were consistent with limitations inherent in a survey study (e.g., ambiguous or undefined terms within the questionnaire, no demographic assessment of participants, and incomplete surveys). Also, many data points were based on subjective answers from participants.

## 6. Conclusion

Because of the rise of food allergies in the pediatric population, particularly in children aged 5 years and younger, preschool staff preparedness to recognize and manage anaphylaxis should be regularly assessed and reinforced. This study illustrates a need and desire among preschool staff for more consistent anaphylaxis education and underscores a significant increase in the comfort level of identifying anaphylaxis and administering an epinephrine autoinjector after a brief educational seminar. Further studies are needed to determine the appropriate interval for and most effective means of education to ensure knowledge retention and sufficient anaphylaxis preparedness.

## Figures and Tables

**Table 1:** Staff characteristics.

Characteristic	Number (%)
Years of providing care (*n* = 169)	
0–5	87 (51)
>5–10	26 (15)
>10–20	30 (18)
>20–30	18 (11)
>30	8 (5)
Ages of children under care^a^ (*n* = 168)	
<6 mo.	50 (30)
6 mo.–2 y	68 (40)
>2–4 y	112 (67)
>4–6 y	98 (58)
>6 y	30 (18)
Children currently under care (*n* = 168)	
0–5	7 (4)
6–10	18 (11)
11–19	89 (53)
20–29	11 (7)
>30	43 (26)

^a^More than 1 response possible. For example, a participant could be caring for children younger than 6 months and children 6 months to 2 years old.

**Table 2:** Staff characteristics, perceived barriers to epinephrine administration, and preintervention assessment.

Characteristic, barrier, or assessment	Overall (*n* = 171)	Director (*n* = 11)	Teacher (*n* =91)	Assistant (*n* = 33)	Other (*n* = 26)	Unspecified (*n* = 10)
Staff characteristic, number (%)						
Currently caring for a child with severe allergies	15/168 (9)	4 (36)	6 (7)	3 (9)	2 (8)	0 (0)
Previous EAI administration	6/170 (4)	1 (9)	4 (4)	0 (0)	1 (4)	0 (0)
Prior EAI training	120 (71)	9 (82)	73 (80)	17 (52)	16 (62)	5/9 (56)
Prior anaphylaxis recognition training	98/171 (57)	9 (82)	58 (64)	13 (39)	12 (46)	6 (60)
Desire for more anaphylaxis education	151/168 (90)	10 (91)	84 (92)	29/32 (91)	24 (92)	4/8 (50)

Perceived barriers to epinephrine administration, number (%)	Overall (*n* = 132)	Director (*n* = 10)	Teacher (*n* = 73)	Assistant (*n* = 23)	Other (*n* = 19)	Unspecified (*n* = 7)

Uncertainty about anaphylaxis recognition	91 (69)	8 (80)	51 (70)	14 (61)	16 (84)	2 (29)
Uncertainty about EAI use	71 (54)	8 (80)	38 (52)	13 (57)	10 (53)	2 (29)
Uncertainty about EAI location	15 (11)	1 (10)	10 (14)	2 (9)	1 (5)	1 (14)
Not allowed to administer EAI	17 (13)	0 (0)	14 (19)	1 (4)	1 (5)	1 (14)
Other	12 (9)	1 (10)	7 (10)	1 (4)	1 (5)	2 (29)
Preintervention staff assessment, mean (SD)						
Anaphylaxis recognition comfort level (*n* = 166)	5.1 (2.4)	4.4 (2.8)	5.2 (2.0)	5.0 (2.3)	4.8 (3.1)	6.5 (2.7)
EAI administration comfort level (*n* = 169)	5.4 (2.8)	5.3 (3.2)	5.7 (2.4)	4.9 (2.8)	5.0 (3.4)	4.7 (3.0)
Number of correctly sequenced steps for EAI administration (*n* = 161)	2.0 (2.1)	1.0 (2.2)	2.3 (2.1)	1.6 (2.2)	1.6 (2.0)	2.0 (2.2)

EAI: epinephrine autoinjector.

**Table 3:** Participant comfort level regarding procedure for EAI administration, before and after the educational intervention.

EAI procedure	Before intervention, mean (SD)	After intervention, mean (SD)	*P* value
Anaphylaxis recognition comfort level (*n* = 166)	5.1 (2.4)	8.7 (1.2)	<.001
EAI administration comfort level (*n* = 169)	5.4 (2.8)	8.8 (1.3)	<.001
Number of correctly sequenced steps for EAI administration (*n* = 161)	3.0 (1.3)	4.2 (1.1)	<.001

EAI: epinephrine autoinjector.
